# Phase I dose-escalation study of tenecteplase, a third-generation fibrinolytic agent, combined with neuronavigation-assisted stereotactic minimally invasive puncture, in patients with acute spontaneous deep cerebral haemorrhage

**DOI:** 10.1136/svn-2025-004389

**Published:** 2025-09-24

**Authors:** Zhiyou Wu, Mingze Wang, Xiudan Bai, Jinyi Tang, Yang Ni, Shaozhi Zhao, Pengqi Wang, Qiheng He, Ran Huo, Yuming Jiao, Duolao Wang, Yong Cao

**Affiliations:** 1 Department of Neurosurgery, Beijing Tiantan Hospital, Capital Medical University, Beijing, China; 2 China National Clinical Research Center for Neurological Diseases, Beijing, China; 3 Department of Clinical Epidemiology and Clinical Trial, Beijing Tiantan Hospital Affiliated to Capital Medical University, Beijing, China; 4 Liverpool School of Tropical Medicine, Liverpool, UK

**Keywords:** Brain, Clinical Trial, Intervention

## Abstract

**Introduction:**

Tenecteplase (TNK) offers logistical advantages in stroke thrombolytic therapy with its single bolus administration compared with alteplase. Moreover, its high specificity for fibrin may contribute to a reduction in haemorrhage complications. However, the safety, tolerability and efficacy of TNK, combined with neuronavigation-assisted stereotactic minimally invasive puncture, in patients with acute spontaneous deep cerebral haemorrhage remain unknown.

**Methods:**

We conducted a prospective, open-label phase I trial in a 3+3 dose escalation design to evaluate the safety and tolerability, and maximum tolerated dose (MTD) of TNK in patients with acute spontaneous basal ganglia or thalamic haemorrhage, with haematoma volumes ranging from 20 to 50 mL, combined with neuronavigation-assisted stereotactic minimally invasive puncture surgery (MIPS). During the dose-escalation phases of the trial, patients received intra-haematoma injection of TNK via a haematoma evacuation catheter every 24 hours after surgery until three doses were administered or any termination criteria were met (residual haematoma ≤10 mL or rebleeding event), with doses ranging from 0.001 to 0.003 and 0.009 mg per mL of haematoma volume. The primary safety endpoint was drug-related rebleeding during the dose escalation phases, while the primary efficacy endpoint was the mean drug-related haematoma clearance per dose.

**Results:**

In total, 12 patients were recruited. No drug-related rebleeding events at any dose escalation phase occurred. By the 24 hours after the last dose, the residual haematoma volume for each patient across all groups was reduced to less than 10 mL. The 0.009 mg TNK dose group achieved the highest mean haematoma clearance of 17.49 mL per dosing. The MTD was 0.009 mg/mL of haematoma volume in the dose escalation phase.

**Discussion and conclusion:**

TNK is well tolerated with encouraging signs of dissolving blood clots. Further exploration of TNK combined with neuronavigation-assisted stereotactic MIPS in patients with acute spontaneous deep cerebral haemorrhage is warranted.

**Trial registration number:**

NCT06668441.

**WHAT IS ALREADY KNOWN ON THIS TOPIC**

- Tenecteplase (TNK), a third-generation thrombolytic agent, possesses increased resistance to inactivation by plasminogen activator inhibitor-1, resulting in greater potency in lysing platelet-rich clots. However, the safety, tolerability and efficacy of TNK, combined with neuronavigation-assisted stereotactic minimally invasive puncture surgery (MIPS), in patients with acute spontaneous deep cerebral haemorrhage remain unknown.

**WHAT THIS STUDY ADDS**

- TNK combined with neuronavigation-assisted stereotactic MIPS in patients with acute spontaneous deep cerebral haemorrhage is well tolerated with encouraging signs of dissolving blood clots. The maximum tolerated dose of TNK is 0.009 mg/mL of haematoma volume in the dose escalation phase.

**HOW THIS STUDY MIGHT AFFECT RESEARCH, PRACTICE OR POLICY**

- Further randomised controlled trials of TNK combined with neuronavigation-assisted stereotactic MIPS in patients with acute spontaneous deep cerebral haemorrhage are warranted.

## Introduction

 Spontaneous intracerebral haemorrhage (ICH) refers to non-traumatic rupture of cerebral blood vessels, leading to blood accumulation within the brain parenchyma, ranking as the second most common subtype of stroke. Its annual incidence is estimated at 12 to 15 cases per 100 000 people, with ICH accounting for 18.8% to 47.6% of all strokes in China.^1^,^4^ The 30-day mortality rate of ICH reaches 35% to 52%, and only approximately 20% of patients regain independence in daily living 6 months after onset,^5^ imposing a significant burden on families and society.^6^

Minimally invasive puncture surgery (MIPS) followed by thrombolysis is favoured for its simplicity and broad applicability, as it effectively aids in haematoma evacuation and decompression in deep acute spontaneous ICHs while minimising iatrogenic injury and maximising neural tissue protection.^7 8^ However, the protection of neurological function through MIPS treatment remains a significant challenge.^9 10^ In the MISTIE III study, good neurological functional outcome result as an mRS 0–3 was 45% in the MIPS followed by alteplase (rt-PA) thrombolysis, while it was 41% in the conservative treatment group, indicating no statistically significant difference between the two groups. Nevertheless, subgroup analysis from the MISTIE III trial revealed that patients undergoing surgical intervention who achieved haematoma reduction to less than 15 mL demonstrated statistically significantly improved functional outcomes compared with those receiving conservative treatment,^11^ which implies that in clinically stable patients, more and rapid removal of intracerebral haematoma may lead to effectively reducing the risk of mortality and improving functional outcomes by lowering intracranial pressure and alleviating haematoma-related irritation.^12 13^ To achieve a better functional outcome, a trial with rapid and large removal of haematoma volumes combined with MIPS is needed.

Tenecteplase (TNK), a third-generation thrombolytic agent, is a genetically engineered medication derived from rt-PA. Its prolonged half-life enables rapid administration through bolus infusion, offering logistic advantages over rt-PA. Moreover, TNK possesses increased resistance to inactivation by plasminogen activator inhibitor-1, resulting in greater potency in lysing platelet-rich clots. Its high specificity for fibrin may contribute to a reduction in haemorrhagic complications, particularly in systemic bleeding.^14^ These features are evidenced both in studies on myocardial infarction and acute ischaemic stroke.^15^,^18^ TNK also demonstrates effective thrombolytic effects to dissolve clots and promote haematoma clearance.^19^ Although TNK is more effective in achieving a clot lysis and avoiding repetitive treatment compared with rt-PA, the safety, tolerability and efficacy of TNK, combined with neuronavigation-assisted stereotactic (NAS) MIPS, in patients with acute spontaneous deep cerebral haemorrhage remain unknown.

We conducted a prospective, open-label, phase I trial in a 3+3 dose escalation design to evaluate the safety and tolerability, and maximum tolerated dose (MTD) or recommended phase II dose (RP2D) of TNK in patients with acute hypertensive basal ganglia or thalamic haemorrhage, with haematoma volumes ranging from 20 to 50 mL combined with NAS MIPS (NAS-TNK). Adverse events and safety monitoring were measured during the dose escalation phases.

## Methods

### Patients

**Figure 1:**
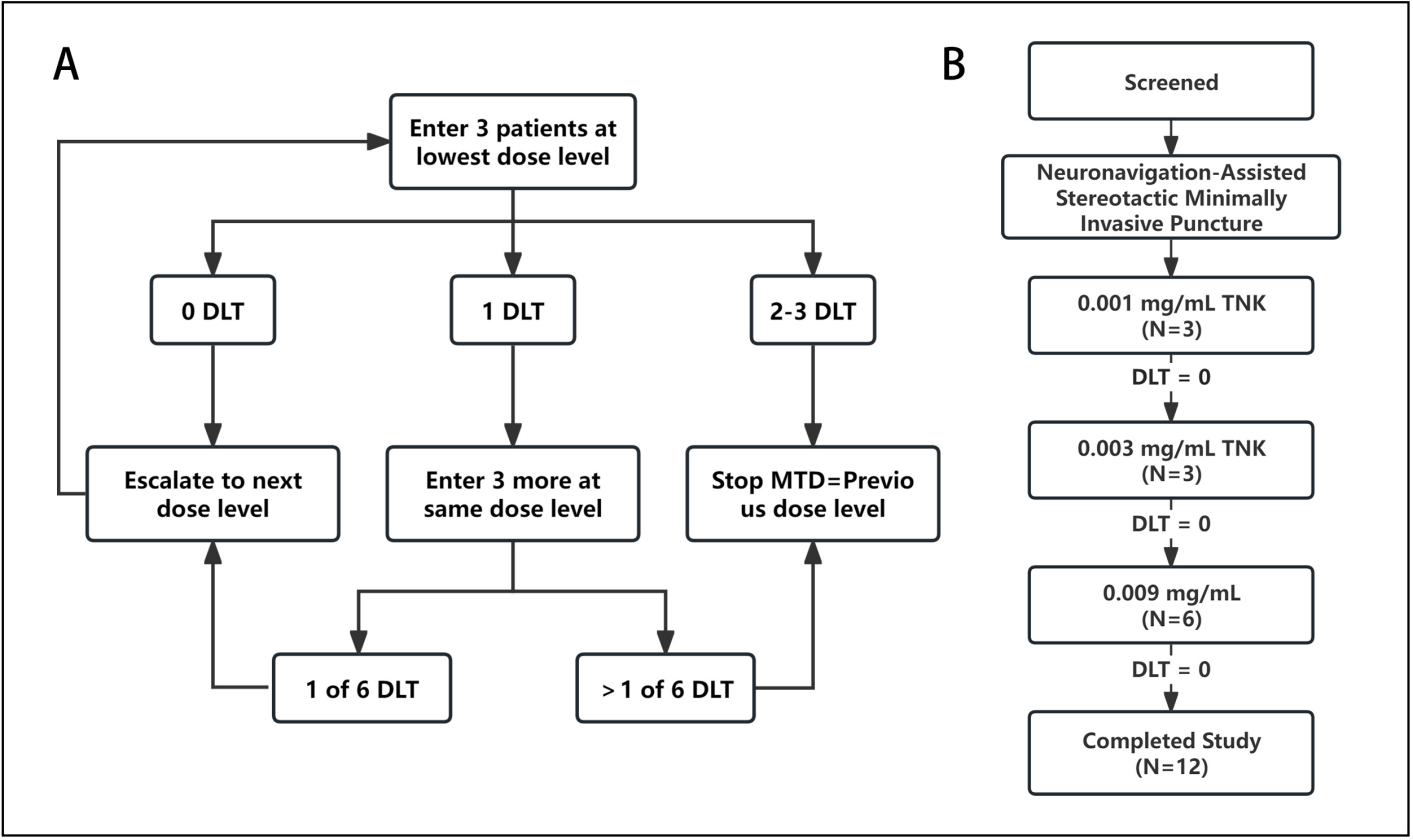
(A) A 3+3 dose escalation design. (B) Flowchart of patient disposition. DLT, dose-limiting toxicity; MTD, maximum tolerated dose; TNK, tenecteplase.

This is a prospective, open-label, phase I trial in a 3+3 dose escalation design (figure 1A) evaluating the safety and tolerability, and MTD or recommended dose of TNK in patients with ICH (20–50 mL) combined with NAS MIPS.

Eligible patients must be screened within 24 hours of symptom onset and show no re-bleeding confirmed by a stability CT scan conducted 6 hours after the initial diagnostic CT. A catheter was inserted by the NAS MIPS. Six hours postsurgery, another stability CT scan should be performed to confirm no rebleeding occurred. Once the haematoma is stabilised, TNK (provided by CSPC Pharmaceutical Group) was administered once daily through the catheter for up to three doses. Eligibility for recruitment should be based on the inclusion and exclusion criteria (Box 1).

Box 1 **Inclusion and exclusion criteria for subjects**

**Inclusion and exclusion criteria**

1. Age ≥18 years and <80 years.
2. Symptoms must have manifested within 24 hours prior to the diagnostic CT scan. Cases with an indeterminate onset time are excluded. For patients who develop symptoms during sleep, the onset time should be determined as the last known awake time.
3. Acute spontaneous deep ICH occurring in the basal ganglia or thalamus, with a volume between 20 and 50 mL as measured by ABC/2 method with radiographic imaging (CT, etc.).
4. Glasgow Coma Scale (GCS) score of 5–14.
5. Stability CT scan done at least 6 hours after diagnostic CT showing clot stability (growth < 5 mL as measured by ABC/2 method).
6. Neuronavigation-assisted stereotactic minimally invasive puncture surgery (MIPS) should be performed within 6 to 24 hours after the diagnostic CT.
7. Systolic blood pressure <180 mm Hg maintained for a duration of 6 hours, documented proximate to the enrolment time point.
8. Prestroke modified Rankin Scale of 0 or 1.

**Exclusion criteria**

1. Lobar or subtentorial haemorrhage, including posterior fossa haemorrhage and cerebellar haemorrhage.
2. Stability CT scan done at least 6 hours after diagnostic CT showing clot instability (growth ≥5 mL as measured by ABC/2 method).
3. Intraventricular haemorrhage necessitating intervention to address mass effect or midline shift attributable to trapped ventricle syndrome secondary to intraventricular haemorrhage-related casting.
4. Haemorrhage attributable to other cerebrovascular pathologies, including but not limited to ruptured aneurysm, arteriovenous malformation, vascular anomalies, moyamoya disease, haemorrhagic transformation of an ischaemic infarct or recurrence of a recent haemorrhage within the past year, as diagnosed through radiographic imaging.
5. Patients presenting with an unstable intracranial mass or progressive intracranial compartment syndrome.
6. Thalamic haemorrhages exhibiting evident extension into the midbrain, accompanied by oculomotor nerve palsy or pupils that are dilated and non-reactive. Other supranuclear gaze abnormalities do not constitute exclusion criteria.
7. Irreversible impairment of brainstem function, characterised by bilateral fixed and dilated pupils, extensor motor posturing and a GCS score of ≤4.
8. Indications for craniotomy in patients include: (1) progressive impairment of consciousness; (2) presence of brain herniation, with signs related to cerebellar tonsil herniation or temporal lobe gyrus herniation; and (3) haematoma located within 1 cm of the cortical surface.
9. CT evidence suggesting a high risk of rebleeding, such as spot sign.
10. Platelet count <100 000/mL; International Normalized Ratio (INR) >1.4.
11. Any irreversible coagulation disorders (eg, haemophilia, von Willebrand disease, use of anticoagulants such as warfarin) or known clotting disorders (eg, hypercoagulable states).
12. Inability to maintain INR ≤ 1.4 using short-acting and long-acting procoagulants (eg, recombinant human coagulation factor VIIa, fresh frozen plasma, vitamin K, etc.).
13. Subjects necessitating long-term anti-coagulation therapy are excluded from participation. Reversal of anticoagulation is permissible for medically stable patients who can feasibly tolerate the short-term risks associated with reversal. Patients must not require Coumadin (warfarin) or other anticoagulants during the initial 30-day period.
14. Prior to the onset of symptoms, anti-coagulants such as dabigatran, apixaban or rivaroxaban, as well as treatments like tirofiban, ticagrelor, cilostazol or clopidogrel, were used.
15. Internal bleeding involving the retroperitoneal, gastrointestinal or genitourinary system or respiratory tract bleeding.
16. Superficial or surface bleeding, observed mainly at vascular puncture and access sites (eg, venous cutdowns, arterial punctures, etc) or site of recent surgical intervention.
17. Positive urine or serum pregnancy test in premenopausal female subjects without a documented history of surgical sterilisation.
18. Allergy/sensitivity to tenecteplase.
19. Engagement in a concurrent interventional clinical investigation or trial. Patients enrolled in observational, natural history or epidemiological studies that do not involve any form of intervention remain eligible.
20. The presence of any concurrent serious illness that could confound outcome assessments, including but not limited to hepatic, renal, gastroenterologic, respiratory, cardiovascular, endocrinological, immunological or haematological disorders.
21. Patients with mechanical heart valves are excluded. The presence of bioprosthetic valve(s) is permissible.
22. Known risk for embolisation, including history of left heart thrombus, mitral stenosis with atrial fibrillation, acute pericarditis or subacute bacterial endocarditis. Atrial fibrillation without mitral stenosis is permitted.
23. Any other condition that, in the investigator’s judgement, would present a significant risk to the subject if the investigational therapy were to be initiated.
24. Active drug or alcohol use or dependence that, in the opinion of the site investigator, would interfere with adherence to study requirements.
25. Patients deemed by the investigator to have unstable conditions that may benefit from other treatments.
26. Patients requesting conservative treatment or standard craniotomy microsurgery treatment.
27. The subject or their legal guardian/representative demonstrates an inability or lack of willingness to provide written informed consent.

### Procedures

Eligible patients will receive the intervention as soon as possible, and they should arrive in the operating room within 8 hours after enrolment. The implementation of NAS-TNK will follow the study surgical manual (online supplemental Supplementary). Throughout the hospitalisation period, all therapeutic management for enrolled subjects will adhere to current clinical guidelines, including those established by the American Heart Association/American Stroke Association.^20^

### Rationale for doses selected

In MISTIE series trials,^11 21^ the alteplase dosage calculation is based on the systemic blood drug concentration: the recommended dosage of alteplase for blood circulation in the guidelines is 0.9 mg/kg for intravenous injection. Since the blood volume per kilogram of body weight is about 75 mL (70–80 mL), it is calculated that the average blood concentration of alteplase is 0.012 mg/mL. In MISTIE series trials,^11 21^ the clot volume of included patients was 30–80 mL; the fixed dosage of haematoma dissolution used was 1 mg (0.012 mg/mL×80 mL) each dosing.

Accordingly, the recommended dosage of TNK for blood circulation is 0.25 mg/kg for intravenous injection. The blood volume per kilogram of body weight in the human body is about 75 mL, and the average blood concentration of TNK should be 0.003 mg/mL:


$$\frac{\frac{0.25mg}{kg}}{\frac{75mL}{kg}}=\frac{0.003mg}{mL}$$

Based on the calculated mean blood concentration of 0.003 mg/mL, we designed three drug escalation dose concentrations of 0.001 mg/mL, 0.003 mg/mL and 0.009 mg/mL. During the dose-escalation phase, patients were sequentially enrolled, with three patients assigned to each dose level. The 0.009 mg dose group could enrol up to 6 patients. The amount of TNK used in a single dose was calculated as the escalation concentration multiplied by the volume of the haematoma (presented on the CT 6-hour post-puncture).


$$Z_{Medication\;Amount}=X_{Dosage\;concentraton}\times Y_{Hematoma\;Volume}$$

### Criteria for termination of tenecteplase (TNK) injection

Termination of TNK injection should meet any of the following conditions: (1) residual haematoma ≤10 mL; (2) rebleeding event (defined as CT demonstrating an increase in ICH by 5 mL); or (3) the patient has received three doses of TNK (treatment endpoint).

### Drainage catheter removal

24 hours after the final drug injection, the drainage catheter will be shut off. The catheter remains closed for at least 24 hours. A follow-up CT scan will be performed 24–36 hours after the shut-off. If there is no ventricular enlargement or other conditions that need surgical intervention, the drainage catheter will be removed. If clinical support for ventricular drainage is required, the catheter may remain in place for over 24 hours.

### Outcome measures

The primary safety outcome was drug-related rebleeding events (dose-limiting toxicity (DLT), defined as a new haemorrhage, is defined as the presence of a lesion with a CT value exceeding 72 Hu and a volume greater than 5 mL in the haematoma cavity and its surrounding area, compared with the previous CT scan). To be classified as drug-related, the rebleeding must satisfy all the following: (1) temporally associated with thrombolytic administration (within 72 hours after dosing) and (2) occurring at the original haemorrhage site. The primary efficacy outcome was the mean drug-related haematoma clearance per dose.

The secondary safety outcomes were symptomatic intracranial haemorrhage, asymptomatic rebleeding, mortality and serious adverse events (SAEs), which were defined as life-threatening events, prolonged or recurrent hospitalisation, disability, dysfunction or abnormalities. The secondary efficacy outcomes included the residual haematoma volume 24 hours after the last dose.

### Postoperative follow-up

Within 7 days postsurgery, symptomatic rebleeding events (defined as a persistent decrease in Glasgow Coma Scale motor score by more than 2 points, with a CT scan showing a new haematoma with a CT value greater than 72 HU and a volume exceeding 5 mL within the haematoma cavity and surrounding area compared with the prior scan) and symptomatic intracranial haemorrhage, asymptomatic rebleeding, mortality and SAEs.

### Sample size

In terms of sample size, the trial was conducted as this was an exploratory study with no formal statistical assessment. However, three, three and six participants in the 0.001 mg/mL, 0.003 mg/mL and 0.009 mg/mL groups were considered adequate to meet the objectives of this study.

### Statistical analysis

Descriptive statistics were produced for outcome variables and for baseline characteristics of patients and safety data by treatment arm. Continuous variables were summarised using the number of observations, mean (SD) or median (IQR), and categorical variables were summarised by the number and percentage. The haematoma volumes in patients were collected at various time points—such as on admission and at 1 day, 2 days, 3 days and 7 days post-treatment—to analyse treatment effects and natural progression.

## Results

### Baseline characteristics

Between November 2024 and January 2025, 42 patients were screened at a neurosurgical centre in China. Of these, 30 patients (71.5%) were screen failures, and 12 participants (28.5%) were successfully enrolled (figure 1B). At the time of study entry, all participants were diagnosed with deep ICH, with haematoma stabilisation within 6 hours. All 12 participants completed the treatment, and none discontinued (figure 1). The baseline characteristics of the participants are summarised (table 1; online supplemental table 1).

**Table 1:** Summary of demographic and baseline characteristics by dosage

Characteristics	0.001 mg	0.003 mg	0.009 mg
Age (years)			
Mean (SD)	59.6 (18.03)	69 (7)	56.5 (13.22)
Sex			
Male n (%)	3 (100%)	2 (66.67%)	5 (83.33%)
BMI (kg/m^2^)			
Mean (SD)	31 (4.78)	25.8 (2.29)	24.5 (6.06)
Hypertension n (%)	2 (66.67%)	2 (66.67%)	3 (50%)
Diabetes mellitus n (%)	0	0	3 (50%)
Hyperlipidaemia n (%)	0	0	3 (50%)
Atrial fibrillation n (%)	0	0	0
National Institutes of Health Stroke Scale (NIHSS) score			
Median	9	14	11.5
Premorbid mRS score (n,%)			
0–1	3 (100%)	3 (100%)	6 (100%)
Onset-to-surgery time (hours)			
Median	29	22	15.5
Haematoma volume change (mL), mean			
Starting volume (mL)	30.49	34.88	31.89
Post-surgery 6 hours (mL)	20.62	29.70	26.95
End-of-treatment volume (mL)	5.53	5.93	6.54
7-day follow-up volume (mL)	3.93	2.24	2.73
Number of drug injections			
Mean (SD)	1.33 (0.58)	1.67 (1.15)	1.16 (0.41)

BMI, body mass index; mRS, modified Rankin Scale.

### Safety outcomes

No rebleeding events or DLT were observed, and no patients withdrew consent. No adverse events such as symptomatic rebleeding occurred in any of the three dosage groups (online supplemental table 2 and figure 1). Across all groups, there were no occurrences of symptomatic intracranial haemorrhage, asymptomatic intracranial rebleeding, patient deaths or SAEs, which were defined as life-threatening events, prolonged or recurrent hospitalisation, disability, dysfunction or abnormalities (online supplemental table 3). No data were missing from the safety outcomes.

### Efficacy outcomes

**Figure 2:**
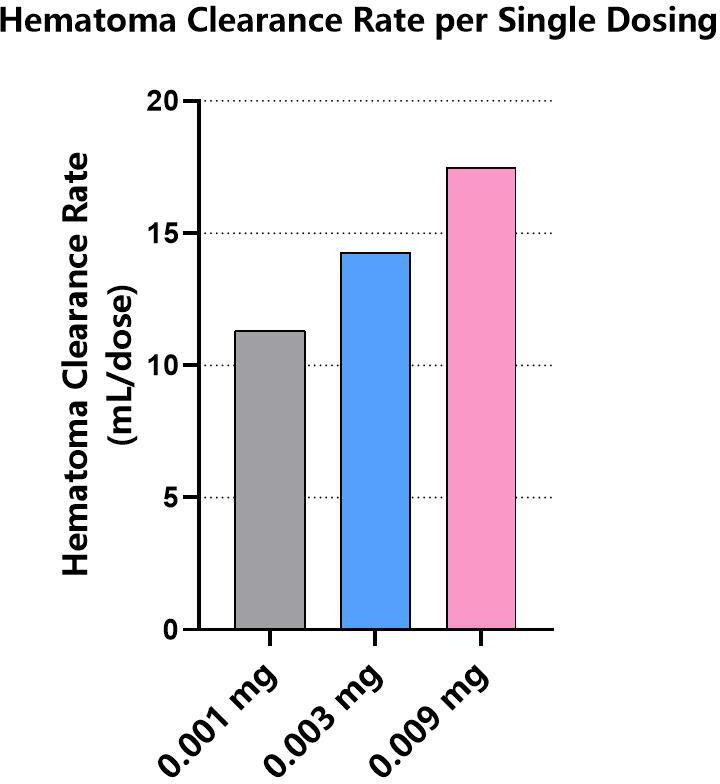
Mean haematoma volume cleared per single dose.

At 24 hours after the last dose, the residual haematoma volume for each patient across all groups was reduced to less than 10 mL (online supplemental table 2; Online supplemental figure 1). As illustrated in figure 2, the mean haematoma volume cleared per single dose associated with TNK showed a dose-dependent increase. Specifically, for the 0.001 mg dosage group, the mean volume cleared was 11.32 mL. This value increased to 14.28 mL for the 0.003 mg dosage group and further to 17.49 mL for the 0.009 mg dosage group. These results suggest a positive dose-response relationship between TNK dosage and haematoma clearance efficiency. The 0.009 mg dose achieved the highest mean haematoma clearance per dose. The MTD was 0.009 mg/mL of haematoma volume in the dose escalation phase.

## Discussion

To the best of our knowledge, this is the first study to present prospective safety data on the use of TNK combined with neuronavigation stereotactic MIPS for treating acute spontaneous deep ICH. The trial also provided data on the efficacy of various TNK doses on clot clearance volume.

In this study, safety outcomes were favourable across all treatment groups, with no adverse events, including symptomatic haemorrhage, asymptomatic rebleeding or mortality within 7 days after surgery. This is particularly encouraging, as the safety profile is a crucial determinant of the feasibility of new therapeutic trials.

Our clinical trial results also showed preliminary evidence that TNK combined with neuronavigation stereotactic MIPS may be effective in evacuating intracerebral haematomas in cases of acute spontaneous deep ICH. Specifically, the NAS-TNK 0.009 treatment group demonstrated a greater volume of haematoma clearance per dose compared with the other two lower-dose groups. Meanwhile, higher concentrations of TNK correlate with more effective haematoma evacuation, suggesting a dose-dependent relationship. Moreover, in the NAS-TNK 0.009 treatment group, almost one administration could achieve residual clot less than 10 mL. Reducing the frequency of administration is not only able to enhance patient compliance, decrease the risk of complications associated with multiple injections but also potentially lower healthcare costs.

In our study, the residual haematoma of all 12 patients reached ≤10 mL at the end of treatment (one patient for 3 TNK dosing, two patients for two dosings and nine patients for one dosing). In contrast, data from the MISTIE III trial demonstrated that patients in the MISTIE group required an average of four thrombolytic administrations, with a mean residual haematoma volume of 16 mL following rt-PA therapy; in our study, the rebleed rate with TNK was 0% and in the MISTIE III, it with rt-PA was 32%.^11^ We have acknowledged that our study was a phase I single centre trial, and the recruited patients were limited, the observed clearance volume and safety outcomes seemed present with improved results.

Our study has three limitations. First, this study was a dose-escalation study primarily focused on assessing the safety and tolerability of TNK combined with NAS minimally invasive puncture in patients with acute spontaneous deep cerebral haemorrhage, rather than establishing efficacy or long-term function recovery. Given its exploratory nature, the primary objective was to identify potential safety and determine an appropriate TNK dose range for future RCT research. We are planning a randomised, open-label, outcome-blinded multicentre trial to investigate the efficiency of the combined surgical modality of NAS minimally invasive puncture combined and TNK (NAS-TNK), for the treatment of acute spontaneous deep ICH. The study will provide the first evidence to guide clinicians in managing the acute spontaneous deep ICH, which remains a serious disease with critical mortality and mobility. Second, we have acknowledged that although the ABC/2 method via CT scan for measuring haemorrhage volume is basic, it is only suitable for calculating regular and small-volume haematomas. Other simplified methods, such as 1/2SH^22^ that are accurate, reliable and rapid methods for calculating haematoma volumes, could be considered for future multicentre trials. However, the ABC/2 method is the most basic practical and widely used method for volume calculation of intracerebral haematoma. It has been used in the high-profile clinical trials, such as the MISTIE series and ENRICH trial. Moreover, we acknowledge that more advanced techniques (eg, artificial intelligence (AI)-assisted segmentation) would provide superior accuracy. However, the AI-assisted more precise imaging techniques or measurements are not popularised and proficient in use.^23^ Meanwhile, this dose-escalation study was for planning a definitive RCT which will be conducted in the multicentres. We are not confident that every centre has consistent and precise imaging techniques or measurements that could be used in this trial. Finally, we did not follow-up patient outcomes in the long term. Future RCTs should comprehensively assess the safety and efficacy of NAS-TNK via longer follow-up time, systematically capturing 30-, 90- and 180-day mRS scores; mortality rates; and neurological complications to enhance clinical benefit evaluation.

## Conclusions

Our research suggested that the use of TNK, particularly at 0.009 mg/mL of haematoma volume, combined with neuronavigation stereotactic MIPS may offer a safe and effective approach for cerebral haematoma clearance. Further randomised controlled trials are necessary to evaluate the long-term functional outcomes of NAS-TNK for patients with acute spontaneous deep ICH.

## Supplementary material

10.1136/svn-2025-004389 online supplemental file 1

## Data Availability

Data are available upon reasonable request.
