# Supplementary material for: Phase I dose-escalation study of tenecteplase, a third-generation fibrinolytic agent, combined with neuronavigation-assisted stereotactic minimally invasive puncture, in patients with acute spontaneous deep cerebral haemorrhage
Source: Stroke Vasc Neurol. 2025 Sep 24;11(3):e004389. doi: 10.1136/svn-2025-004389 (PMC13347912; doi:10.1136/svn-2025-004389)
Supplement: online supplemental file 1 [file svn-11-3-s001.docx]

**A phase I dose-escalation study of tenecteplase, a third-generation fibrinolytic agent, combined with neuronavigation-assisted stereotactic minimally invasive puncture, in patients with acute spontaneous deep cerebral hemorrhage**

**Supplementary Table 1.**  List of baseline characteristics of enrolled patients

**Supplementary Table 2.**  List of CT hematoma volume and GCS outcomes by dosage and time

**Supplementary Figure 1.** Imaging and hematoma volume change chart for patients in the TNK group

**Supplementary Table 3.**  Safety outcomes by dosage

**Supplementary Figure 2.**  TNK-related hematoma changes by dosage

**Clinical manuals**

| **Supplementary Table 1.** List of baseline characteristics of enrolled patients | | | | | | | | | | | | | | | |
| --- | --- | --- | --- | --- | --- | --- | --- | --- | --- | --- | --- | --- | --- | --- | --- |
| **Grouping** | **No.** | **Age (years)/sex** | **BMI (kg/m²)** | **Preop GCS Score** | **NIHSS** | **Premorbid mRS score** | **Hypertension** | **Diabetes mellitus** | **Hyperlipidaemia** | **Atrial fibrillation** | **Location/Side** | **IVH** | **dCT** | **Stable CT** | **OST (h)** |
| 0.001 mg | 1 | 41/M | 29.73 | 13 | 9 | 0 | N | Y | N | N | BG/L | N | 30.66 | 32.48 | 32 |
| 0.001 mg | 2 | 77/M | 36.3 | 14 | 9 | 0 | N | Y | N | N | BG/R | N | 24.79 | 29.29 | 16 |
| 0.001 mg | 3 | 61/M | 26.99 | 14 | 9 | 0 | N | N | N | N | BG/R | N | 30.14 | 29.69 | 29 |
| 0.003 mg | 4 | 69/M | 25.95 | 12 | 9 | 0 | N | Y | N | N | BG/R | N | 20.84 | 23.29 | 27 |
| 0.003 mg | 5 | 76/M | 28.01 | 10 | 14 | 0 | N | Y | N | N | BG/R | N | 44.25 | 48.59 | 22 |
| 0.003 mg | 6 | 62/F | 23.44 | 14 | 14 | 0 | N | N | N | N | BG/R | N | 30.1 | 32.76 | 17 |
| 0.009 mg | 7 | 46/M | 22.86 | 12 | 14 | 0 | N | N | N | N | BG/L | N | 27.19 | 30.75 | 18 |
| 0.009 mg | 8 | 67/M | 19.59 | 13 | 14 | 0 | N | Y | N | N | Thal/R | Y | 29.56 | 29.54 | 15 |
| 0.009 mg | 9 | 52/F | 24.61 | 11 | 13 | 0 | N | N | N | N | BG/L | N | 32.75 | 35.54 | 11 |
| 0.009 mg | 10 | 61/M | 22.6 | 14 | 10 | 0 | Y | N | Y | N | BG/R | N | 32 | 32 | 39 |
| 0.009 mg | 11 | 39/M | 36.3 | 14 | 8 | 0 | Y | Y | Y | N | BG/R | N | 21.99 | 22 | 16 |
| 0.009 mg | 12 | 74/M | 20.76 | 14 | 8 | 0 | Y | Y | Y | N | BG/L | N | 40 | 41.5 | 11 |
| No. Number of Patients; Preop GCS Score Preoperative GCS Score; BMI Body Mass Index; IVH Intraventricular Hemorrhage; BG Basal Ganglia; Thal Thalamus; dCT Diagnostic CT; OST Onset-to-Surgery Time: duration from onset of symptoms to surgical intervention; TNK Tenecteplase; CT Computed tomography; BG Basal Ganglia; Thal Thalamus; L Left; R right; | | | | | | | | | | | | | | | |

| **Supplementary Table 2.**  List of CT hematoma volume and GCS outcomes by dosage and time | | | | | | | | | | | |
| --- | --- | --- | --- | --- | --- | --- | --- | --- | --- | --- | --- |
| **Dosage** | **No.** | **CT 6h Postop** | **1st dosing (mg)** | **CT 24 h post 1st dosing** | **CT 48 h post 1st dosing** | **CT 72 h post 1st dosing** | **CT 96 h post 1st dosing** | **CT 120h post 1st dosing** | **7-10 day CT** | **Discharge GCS Score** | **No. of Doses.** |
| 0.001mg | 1 | 27.06 | 0.027 | **8.6** | 4.7 | **11.02** |  |  | 10.5 | 13 | 1 |
| 0.001mg | 2 | 22.09 | 0.022 | 11.91 | **6.55** | 6.39 | **6.39** |  | 0 | 14 | 2 |
| 0.001mg | 3 | 12.72 | 0.012 | **1.45** | 1.72 | **2.1** |  |  | 1.3 | 14 | 1 |
| 0.003 mg | 4 | 14.82 | 0.044 | **1.125** | 1.14 | **1.38** |  |  | 0 | 12 | 1 |
| 0.003 mg | 5 | 42.99 | 0.128 | 23.78 | 15.01 | **9.62** | 7.2 | **5.52** | 5.52 | 10 | 3 |
| 0.003 mg | 6 | 31.36 | 0.094 | **7.05** | 3.15 | **1.47** |  |  | 1.2 | 14 | 1 |
| 0.009 mg | 7 | 29.64 | 0.266 | 16.5 | **6.6** | 3.4 | **2.58** |  | 1.2 | 12 | 2 |
| 0.009 mg | 8 | 23.2 | 0.208 | **8.79** | 4.68 | **3.52** |  |  | 0 | 13 | 1 |
| 0.009 mg | 9 | 26.22 | 0.236 | **5.56** | 5.56 | **6.14** |  |  | 0 | 11 | 1 |
| 0.009 mg | 10 | 33.12 | 0.298 | **6.1** | 5.44 | **4.86** |  |  | 1.98 | 14 | 1 |
| 0.009 mg | 11 | 20.7 | 0.186 | **2.66** | 1.17 | **1.15** |  |  | 1.11 | 14 | 1 |
| 0.009 mg | 12 | 28.82 | 0.259 | **9.55** | 9.42 | **11.83** |  |  | 12.11 | 14 | 1 |
| No. Number of Patients; OST Onset-to-Surgery Time; TNK Tenecteplase; CT Computed tomography; CT 6h Postop CT Scan 6 Hours Postoperative; 1st dosing First dosing; BG Basal Ganglia; Thal Thalamus; L Left; R right; **Red** indicates that the patient has met the criteria for discontinuation; **Green** indicates the hematoma volume 24 hours after catheter removal, which also corresponds to 72 hours after the last dose for the patient. | | | | | | | | | | | |

**Supplementary Figure 1.** Imaging and hematoma volume change chart for patients in the TNK group


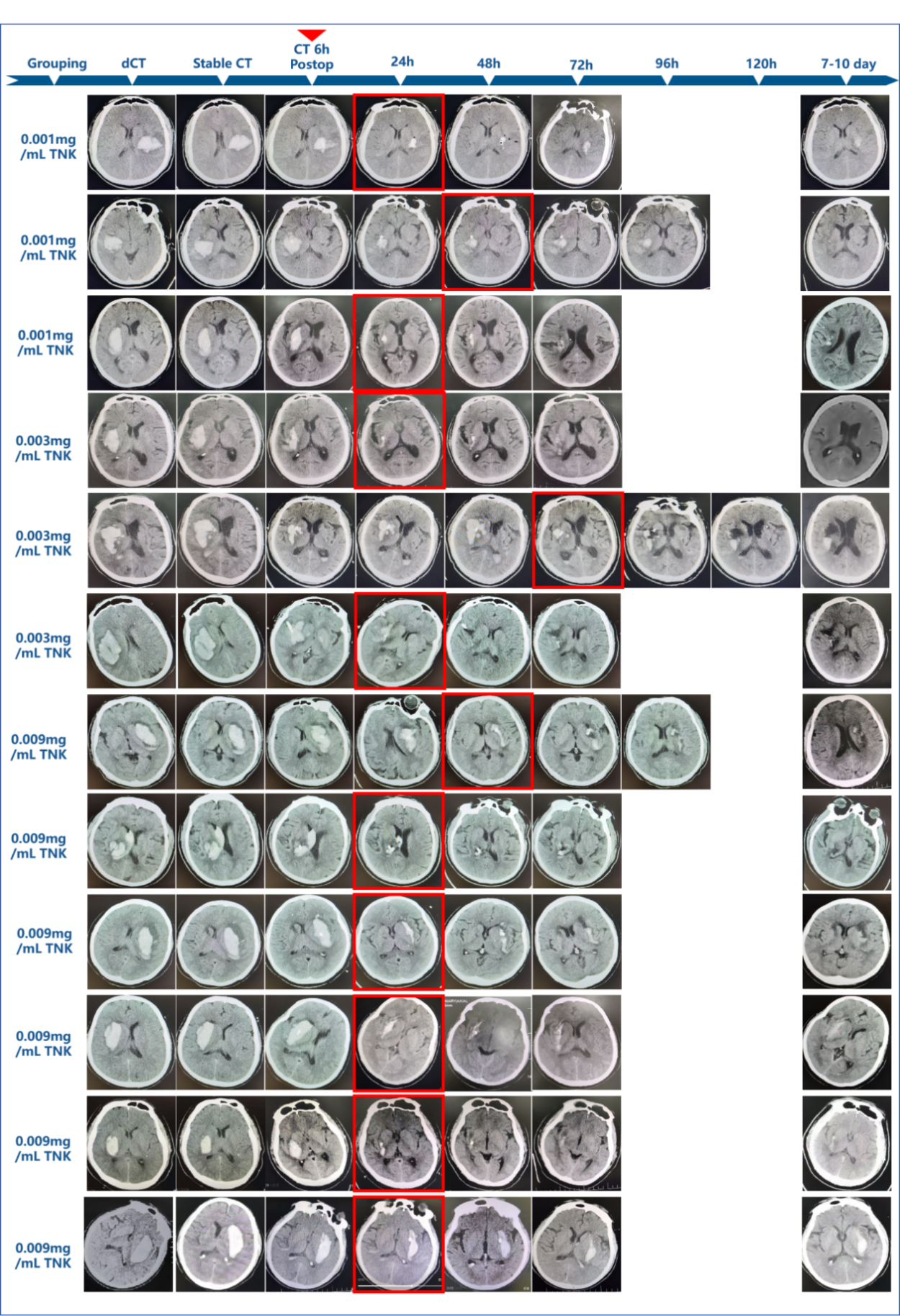


dCT Diagnostic CT; CT 6h Postop CT Scan 6 Hours Postoperative; "▼"indicates the first administration of TNK medication via a drainage tube; TNK Tenecteplase; "**□**" indicates that the patient has met the criteria for termination of TNK injection;

**Supplementary Table 3.** Safety outcomes by dosage

|  | **TNK** | | |
| --- | --- | --- | --- |
|  | **0.001 mg**  **(n=3)** | **0.003 mg**  **(n=3)** | **0.009 mg**  **(n=6)** |
| Symptomatic ICH at 72 hours | 0 | 0 | 0 |
| Death | 0 | 0 | 0 |
| Asymptomatic intracranial haemorrhage | 0 | 0 | 0 |
| Other extracranial bleeding | 0 | 0 | 0 |
| Serious adverse events | 0 | 0 | 0 |

TNK Tenecteplase;

**Supplementary Figure 2.** TNK-related hematoma changes by dosage


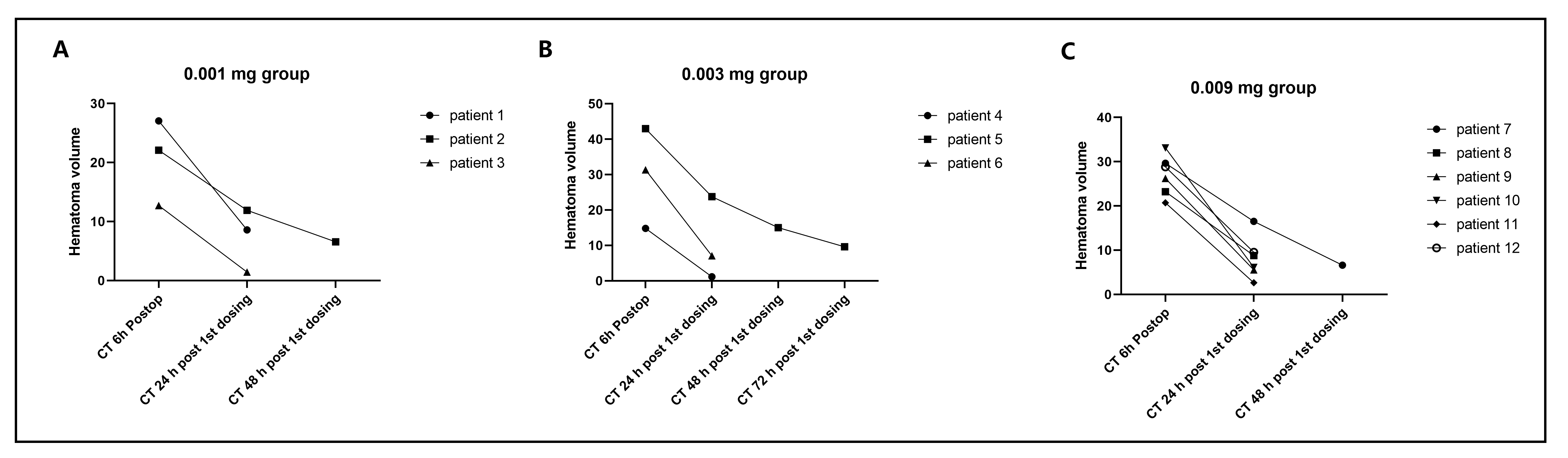


**Clinical manuals**

Surgical Manual

Neurosurgeons with specialized surgical training will perform NAS-TNK therapy to evacuate intracerebral clots, strictly adhering to the established protocol.

Timing of surgery

Considering the existing preclinical biological evidence, early intervention will benefit the prognosis of patients with intracerebral hemorrhage. The study requires patients to be brought to the operating room within 8 hours after enrollment.

Patient position

Patients should be positioned supine with a slight forward tilt of the head. Depending on the requirements of the navigation instrument, the head can be stabilized with or without a head-holder. When using an optical neuronavigation system, slightly turning the face toward the detector may improve the accuracy of navigation registration. In other cases, the head should be positioned according to the specific requirements of the procedure. Additionally, the planned puncture point should be chosen to ensure ease of access and manipulation.

Anesthetic plan

The surgical procedure is performed in a sterile operating room under general anesthesia, using either endotracheal intubation or laryngeal mask insertion. Blood pressure is strictly monitored during the procedure, with a target systolic blood pressure of less than 140 mmHg. For patients with favorable pre-induction neurological conditions, immediate extubation following surgery is recommended.

Neuronavigation-assisted stereotactic minimally invasive puncture surgery

Surgical planning

Preoperative planning can be performed on a standalone workstation or integrated within the navigation system. High-resolution preoperative images, including cranial CT and CTA scans with a slice thickness of less than 1 mm, and an adequate scanning range (extending from the apex of the nose to the calvaria, or at least from the zygoma to the calvaria), are essential for meticulous planning of a safe and effective trajectory. Three-dimensional reconstructions of the skull should be utilized as a reference for selecting the entry point, while the hematoma should guide the selection of the target point. The entry point should be located within a range extending from the coronal suture to 5 cm anterior to it, 2 cm lateral to the midline on the hematoma side, and up to the temporal line. The puncture target point is defined as the centroid of the hematoma, located at the intersection of the sagittal, transverse, and coronal planes in the layer with the largest hematoma area. The selected puncture point should be as close as possible to the target point while avoiding blood vessels along the puncture path. The trajectory should be further refined using vascular imaging in the probe view to minimize the risk of injuring arterial or venous vessels. [1].

Registration

Important landmarks, such as the eyes, nose, cheeks, and scalp, should remain uncovered and free from deformation. Registration can be performed using different methods, depending on the neuronavigation system and the surgeon's preference. The accuracy of registration should be within 1 mm. This accuracy should be verified not only by the error value displayed on the system but also through practical testing with the probe at the following anatomical points: the nasion, inner canthus, external canthus, external acoustic meatus, and the apex of the nose (if accessible). The on-site surgeon must ensure that the entry point is located within the hairline or make adjustments as needed.

Operative Setup

Most navigation systems consist of two main components: a computer workstation (with or without a touchscreen monitor) and an optical or electromagnetic detector. Additional attachments typically include a sterilized probe, a reference ring with navigation spheres or an electromagnetic tracker, a manual or stereotactic device, and its supporting arm. The arrangement of these components depends on the operating mode of the navigation system. For optical navigation systems, which require the use of a head-holder, a one-to-two adapter is commonly used to secure both the reference ring and the supporting arm of the stereotactic device to the C-frame. It is recommended to position the supporting arm and detector on the same (operative) side to ensure adequate range of motion for the stereotactic device and supporting arm and to provide an optimal field of view for detection. In contrast, for electromagnetic navigation systems, sufficient distance between the detector and the patient, along with minimal skin shifting under the tracker, must be maintained.

Intraoperative Use

It is recommended to intermittently check and adjust any deviation between the actual manipulation and the planned trajectory, particularly when approaching the entry point. Verifying the incision and burr-hole locations can assist in making necessary corrections. In certain cases, the entry point may be positioned within the inner cortical bone of the skull to minimize errors caused by the orientation of the burr-hole. Before incising the dura mater, the stereotactic device can be mounted to verify the puncture point and trajectory one final time.

Puncture

After positioning with the manual or neuronavigation stereotactic device, a small incision is made. For optical navigation systems, the depth of the puncture catheter should follow the planned trajectory. For electromagnetic navigation systems, the tip of the puncture catheter can be tracked using a flexible stylet. Once the target location is reached, the drainage tube is secured subcutaneously at a position away from the incision and connected to a three-way valve and a drainage bag. Postoperatively, patients are transferred to a specialized ward with a multidisciplinary team (stroke unit) or a neuro-intensive care unit for treatment, where vital signs are closely monitored. Standard medical care is administered in accordance with guideline recommendations.

The stability of the hematoma was examined

A follow-up CT scan was performed 6 hours post-puncture to evaluate the position of the drainage tube, the hematoma, and any new bleeding. If the clot volume measured on this initial follow-up CT scan increases by 5 mL or more, a second follow-up CT scan is allowed at least 12 hours later. Additional scans may be performed every 12 hours as needed to monitor for stability, up to 72 hours after the diagnostic CT scan. Once clot stability is confirmed—defined as growth of less than 5 mL between two consecutive CT scans (measured by the ABC/2 method), and the hematoma size remains ≤ 60 mL—the patient becomes eligible to begin TNK therapy for hematoma lysis. However, if bleeding persists and the hematoma volume exceeds 60 mL or remains unstable beyond 72 hours post-diagnostic CT scan, alternative surgical intervention for hematoma evacuation should be performed in accordance with standard surgical principles (Figure 2).

Tenecteplase drug injection

The prescribed dose of tenecteplase (TNK) will be dissolved in 1-3 mL of 0.9% sodium chloride solution and administered into the hematoma cavity through the drainage tube. The tube will be flushed with 3 mL of normal saline, then shut-off for 2 hours to facilitate drug-clot interaction, and then turn on to allow gravity drainage. The medication will be administered once every 24 hours, for a maximum of three doses. The following conditions will necessitate early termination of TNK injection: (1) residual hematoma ≤10 mL; (2) rebleeding event (defined as CT-demonstrated the intracerebral haemorrhage increases by 5 mL).

Medical Manual

All subjects will be treated according to the Clinical Standardization Guidelines (CSG). The CSG has been adapted based on the 2022 AHA/ASA guidelines for the management of spontaneous intracerebral hemorrhage [1]. Whenever clinically feasible, the CSG should be followed, as they provide a framework for the care of these subjects. The attending physician has ultimate responsibility and discretion in treating the subjects. The physician will use their best judgment in accordance with the specific clinical situation and good clinical practice (GCP) when providing treatment.

Blood Pressure Control

Blood pressure stability is defined as a sustained systolic blood pressure (SBP) of less than 180 mm Hg for at least 6 hours prior to enrollment. During the 6-hour monitoring period, both systolic and diastolic blood pressures should be documented as source data in the medical record. Blood pressure management after enrollment (according to the 2022 AHA/ASA guidelines) includes: for patients with an SBP greater than 220 mm Hg, aggressive blood pressure reduction should be implemented under continuous monitoring to achieve an SBP of 180 mm Hg; for patients with an SBP between 150-220 mm Hg, the target is to reduce the SBP to the range of 130-140 mm Hg; for those with an SBP greater than 150 mm Hg, efforts should be made to avoid reducing the SBP below 130 mm Hg. The process of blood pressure reduction requires continuous monitoring, and careful control of the infusion or pump rates is necessary to ensure a steady and gradual reduction in blood pressure.

Management of intracranial pressure

Intracranial pressure management involves measures such as elevating the head position and administering osmotic agents. The primary treatment for osmotic therapy is intravenous infusion of 20% mannitol, which may be used in conjunction with furosemide, glycerol, hypertonic saline, and/or albumin when necessary.

Neurological monitoring

Neurological status will be assessed using the Glasgow Coma Scale (GCS) every 4 hours. Neurological deterioration is defined as a decrease of more than 2 points on the motor scale of the GCS in a patient who is not sedated, sustained for a duration of 8 hours.

Respiratory Care

Respiratory care must ensure adequate oxygenation without compromising the airway and achieving complete lung inflation. The patient should breathe room air, maintaining an oxygen saturation level of ≥90%, or receive supplemental oxygen through a mask with an oxygen concentration of 28% or lower.

Nutritional Support

Nutritional support should provide ≥30 kcal/kg and 1.5 g of protein/kg intake. Nutritional support may be delivered through the least invasive methods necessary, and full nutritional support should be achieved within 7 days of the onset of illness.

Withdrawal of Technological Support

Aggressive care will be maintained for at least 72 hours and do-not-resuscitate (DNR) orders will be delayed for a minimum of 48 hours following admission. Participants with pre-existing DNR orders or advanced directives that would reasonably restrict aggressive care are not eligible for the trial.

Imaging Examination

Besides the CT scans 24 hours after dosing, the patient is scheduled to undergo a follow-up CT scan for 7 to 10 days after surgery when necessary. This imaging procedure is intended to assess the surgical site for any potential complications, evaluate the progress of healing, and ensure that there are no unexpected developments. By conducting this follow-up scan within this timeframe, healthcare providers can make informed decisions about the patient's postoperative care and adjust treatment plans as necessary.

Reference

1. Greenberg, S.M., et al., 2022 Guideline for the Management of Patients With Spontaneous Intracerebral Hemorrhage: A Guideline From the American Heart Association/American Stroke Association. Stroke, 2022. 53 (7): p. e282-e361.


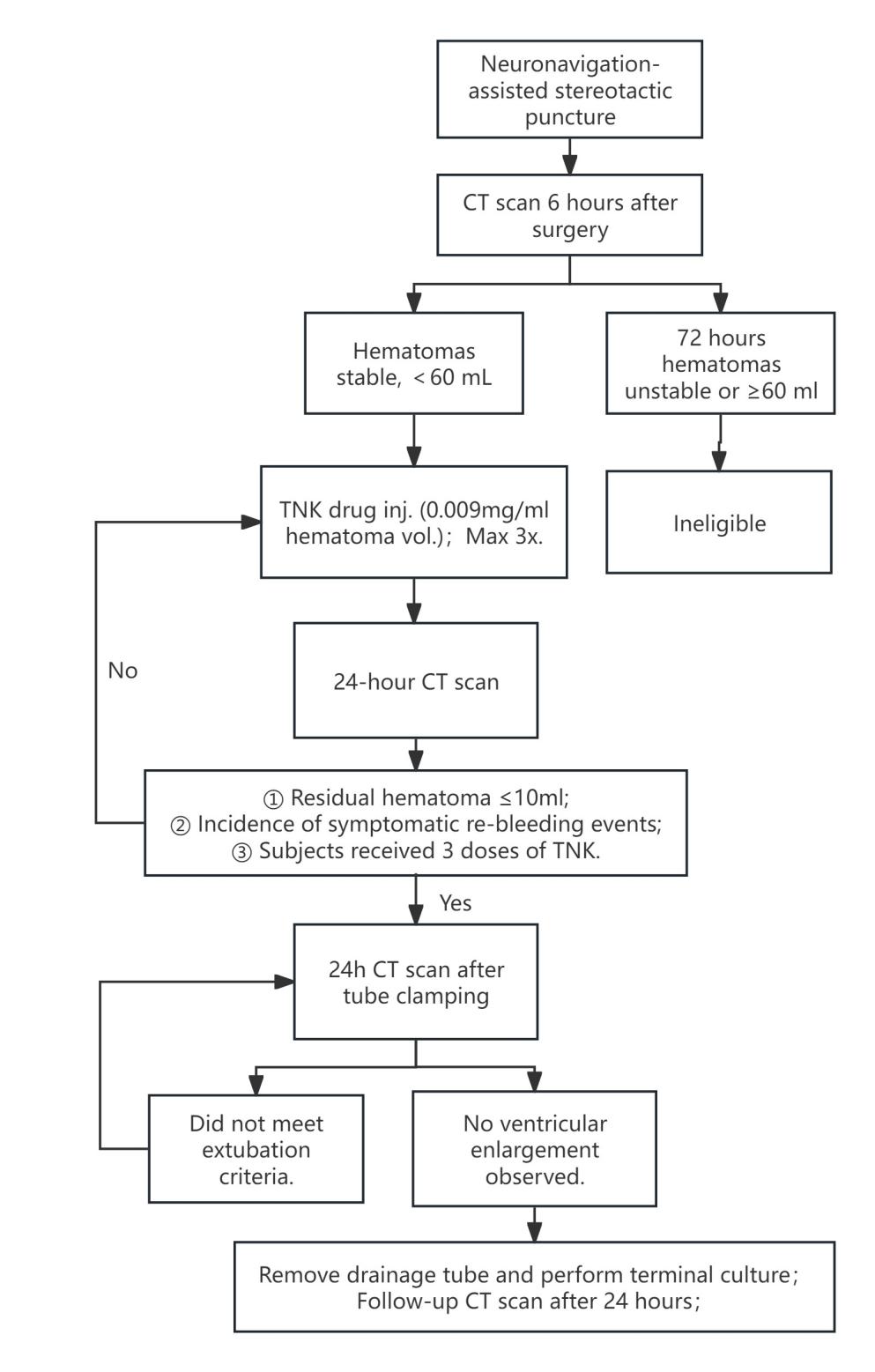


**Figure 2.** NAS-TNK Group Flowchart
